# Total and High Molecular Weight Adiponectin Expression Is Decreased in Patients with Common Variable Immunodeficiency: Correlation with Ig Replacement Therapy

**DOI:** 10.3389/fimmu.2017.00895

**Published:** 2017-07-31

**Authors:** Antonio Pecoraro, Ersilia Nigro, Rita Polito, Maria Ludovica Monaco, Olga Scudiero, Ilaria Mormile, Azzurra Cesoni Marcelli, Mario Capasso, Francesco Habetswallner, Arturo Genovese, Aurora Daniele, Giuseppe Spadaro

**Affiliations:** ^1^Department of Translational Medical Sciences, Allergy and Clinical Immunology, University of Naples Federico II, Naples, Italy; ^2^CEINGE-Biotecnologie Avanzate Scarl, Napoli, Italy; ^3^Dipartimento di Scienze e Tecnologie Ambientali Biologiche Farmaceutiche, Università degli Studi della Campania “Luigi Vanvitelli”, Caserta, Italy; ^4^Dipartimento di Medicina Molecolare e Biotecnologie Mediche, Università di Napoli Federico II, Napoli, Italy

**Keywords:** common variable immunodeficiency, adiponectin, high molecular weight oligomers, immunoglobulin, adipose tissue, intravenous immunoglobulin, immunoglobulin replacement therapy

## Abstract

Adiponectin (Acrp30) is an adipokine widely studied for its beneficial metabolic properties. It circulates as low molecular weight (LMW), medium molecular weight (MMW), and high molecular weight (HMW) oligomers. The latter exerts the most potent biological effects. Acrp30 attracted renewed interest with the finding that it was associated with the development and progression of immune disorders. The mechanisms underlying this association and the role of Acrp30 in the pathophysiology of immune-mediated conditions remain unknown. Common variable immunodeficiency (CVID) is a primary immunodeficiency characterized by chronic activation of the immune system, impaired antibody production, and imbalanced cytokine production. In the attempt to shed light on the expression of Acrp30 in CVID, we: (a) investigated total Acrp30 and its oligomerization state in CVID patients undergoing maintenance Ig replacement therapy; (b) assessed the effects of Ig replacement therapy on Acrp30 expression in treatment-naïve CVID patients, namely, patients not treated before diagnosis, before and after the first Ig administration; and (c) evaluated the correlation between Acrp30 levels and clinical phenotypes of the disease. As controls, we analyzed healthy subjects and patients affected by a non-immunodeficiency chronic inflammatory demyelinating polyneuropathy (CIDP), before and after Ig infusion. We found that total Acrp30 and HMW oligomers were decreased in CVID but not in CIDP patients versus controls. Moreover, Acrp30 levels were correlated with IgA levels and were associated with two CVID phenotypes, namely, autoimmune cytopenia and enteropathy. Receiver operating characteristic curve analysis indicated that Acrp30 modulation is specific for CVID patients. Acrp30 and HMW levels quickly and dramatically increased after Ig infusion only in eight treatment-naïve CVID patients but not in five CIDP patients. This finding indicates that Ig administration *per se* is not able to induce an increase of Acrp30, but the specific cellular and/or molecular background proper of CVID seems to be essential. In conclusion, our data indicate that Acrp30 is specifically related to CVID activity. Further studies are required to understand the biological role of Acrp30 and its possible use as disease biomarker in CVID.

## Introduction

Common variable immunodeficiency (CVID) is a primary immunodeficiency characterized by reduced serum levels of IgG, IgA, and/or IgM, which results in impaired antibody production. CVID is one of the most frequent primary immunodeficiencies in adults and its prevalence ranges from 1:10,000 to 1:50,000 (1). It is generally diagnosed between the ages of 20 and 40 years (1). CVID patients can be divided into four clinical phenotypes: (1) no CVID-related complications besides infections; (2) cytopenias (thrombocytopenia, autoimmune hemolytic anemia or neutropenia); (3) polyclonal lymphoproliferation (granuloma, lymphocytic interstitial pneumonia, persistent unexplained lymphadenopathy); and (4) unexplained persistent enteropathy (2–4). An altered cytokine profile and abnormalities in immune cellular subpopulations consistent with a substantial chronic inflammatory condition have been reported in CVID patients (5, 6); however, the mechanisms underlying the chronic immune activation associated with CVID remain largely obscure.

Beyond the well-known impairment of B cell function, the spectrum of cytokines upregulated in CVID patients seems to reflect ongoing activation of several myeloid cells mainly monocytes, macrophages, and neutrophils (5, 6). White adipose tissue is an important site for the establishment of inflammation consequent to secretion of the biologically active mediators, adipokines (7–10). One of these, namely adiponectin (Acrp30), circulates in serum as complexes of different molecular weight: low molecular weight (LMW), medium molecular weight (MMW), and high molecular weight (HMW) (11, 12). Interestingly, HMW Acrp30 oligomers elicit more potent biological effects than do the other two oligomers (12). Acrp30 expression is low in such metabolic disorders as obesity, type 2 diabetes mellitus, and obstructive sleep apnea syndrome (13–16). In patients affected by these diseases, Acrp30 is often negatively associated with the inflammatory markers tumor necrosis factor α (TNF-α), interleukin 6 (IL-6), and C reactive protein. Conversely, Acrp30 is overexpressed in autoimmune diseases, mainly rheumatoid arthritis and systemic lupus erythematosus, and in chronic obstructive pulmonary disease, which are diseases characterized by a chronic inflammatory status (1, 17). Neither the causes of Acrp30 upregulation nor the role it plays in these disorders is known. In addition, Acrp30 receptors are expressed on the surface of peripheral blood mononuclear cells, which is consistent with functional immunomodulatory signaling (18–21). Acrp30 is also an important regulator of macrophage proliferation, plasticity, and function in inflammation and its related metabolic disorders (22–24). Acrp30 inhibits the growth of myelomonocytic progenitors (22) and downregulates the inflammatory responses involving TNF-α (10, 25–29). In addition, Acrp30 oligomers may exert different functions, for example HMW, but not LMW. Acrp30 enhances IL-6 production in primary human monocytes (30, 31). Some adipokines including leptin, were recently found to be differentially modulated in CVID patients versus control subjects (30, 32–35).

The aims of the present study were to: (i) analyze Acrp30 expression levels and its oligomerization state in a large cohort of CVID patients in maintenance Ig replacement therapy; (ii) determine whether Acrp30 fluctuations are related to different clinical phenotypes; (iii) investigate the role of Acrp30 in immunodeficiency by monitoring the levels of this adipokine in eight treatment-naïve CVID patients (i.e., patients not treated before diagnosis) before and after the first Ig replacement therapy administration versus. As controls, we measured Acrp30 levels in healthy subjects and in patients affected by chronic inflammatory demyelinating polyneuropathy (CIDP) before and after Ig infusion therapy versus five CIPD patients before and after Ig infusion.

## Materials and Methods

### Recruitment of Subjects

We recruited 52 patients in maintenance treatment with Ig (28 men, 24 women) and 8 (4 men and 4 women) treatment-naïve patients at the time of CVD diagnosis based on the 2014 European Society for Immunodeficiencies diagnostic criteria of 2014, from the Division of Allergy and Clinical Immunology of the Department of Translational Medical Sciences, University of Naples Federico II. In addition, we recruited five patients (three men, two women) with a diagnosis of CIDP from the Azienda Ospedaliera A. Cardarelli.

From the medical files of CVID patients, we recorded serum Ig levels, T cell count, and B cell subsets at diagnosis, clinical history of recurrent infections, chronic diarrhea, bronchiectasis, autoimmune diseases (autoimmune hemolytic anemia, immune thrombocytopenia, neutropenia), polyclonal lymphoproliferation (splenomegaly, lymphadenopathy, and granulomatous disease), and malignancies.

Common variable immunodeficiency patients received continuous Ig replacement therapy (36 patients received IgVena^®^/Kedrion, 6 patients Flebogamma^®^/Grifols, 5 patients Kiovig^®^/Baxalta, 2 patients Gammagard^®^/Baxalta, 2 patients Octagam^®^/Octapharma, and 1 patient Privigen^®^/Behring) (0.4 g/kg/month) at intervals of 3 weeks to maintain IgG trough levels above 600 mg/dl (768 ± 87 mg/dl). There were no familial cases in the control group or among CVID patients. Treatment-naïve patients received intravenous Ig immunomodulating therapy (IgVena^®^ 50 mg/ml by Kedrion S.p.A. at the dose of 0.4 g/kg/21 days). Five patients with CIDP receiving intravenous immunoglobulin (IVIG) immunomodulating therapy (IgVena^®^ 50 mg/ml by Kedrion S.p.A. at the dose of 0.4 g/kg/day for 5 consecutive days) were collected. All CIDP patients had previously received IVIG immunomodulating therapy. Purity at 99% of Ig for the replacement therapy was guaranteed by Kedrion S.p.A. Fifty-four healthy volunteers from the CEINGE staff (27 men, 27 women), age-, body weight- and body mass index (BMI)-matched with the 52 CVID patients served as control group.

The research protocol was approved by the Ethics Committee of the School of Medicine, University of Naples “Federico II” and was conducted in accordance with the principles of the Helsinki II Declaration. Written informed consent was obtained from all participants.

### Anthropometric and Metabolic Measurements

The height and weight of patients were measured using standard techniques and the BMI was calculated as body weight (kg)/height^2^ (m^2^). Samples of serum were collected from all patients and from the eight treatment-naïve patients before the first Ig replacement administration (0.4 g/kg) and 24 h, 7 days, 14 days, and 21 days thereafter. Serum samples were collected from patients with CIDP before Ig therapy and 24 h and 7 days thereafter (0.4 g/kg). Biochemical measurements are reported in Table S1 in Supplementary Material. We verified the purity of the preparation by sodium dodecyl sulfate-polyacrylamide gel electrophoresis (SDS-PAGE) analysis stained with Coomassie brilliant blue and by ensuring the absence of Acrp30 in Ig solutions through western blotting with two Acrp30 antibodies (Custom antibody; PRIMM, Milan, Italy; Cell Signaling Technology, MA) (data not shown). Biochemical measurements are reported in Table 1 and Table S1 in Supplementary Material. Total Acrp30 was measured by enzyme-linked immunosorbent assay (ELISA) method as previously reported (32).

**Table 1:** Clinical and biochemical features of common variable immunodeficiency (CVID) patients and controls.

Parameters	CVID patients *N* = 52	Mean	SD	Controls *N* = 54	Mean	SD	*p*-Value
Sex male/female	28/24	–	–	27/27	–	–	0.69
Age (years)	52	48.7	16.9	54	49.3	16.3	0.704
Weight (kg)	52	68.0	14.6	51	69.5	12.7	0.345
Body mass index (kg/m^2^)	52	24.7	4.0	54	23.9	3.2	0.383
Total cholesterol (mg/dl)	52	173.3	39.8	50	197.4	45.9	**0.015**
Tryglicerides (mg/dl)	52	103.6	44.3	51	99.0	46.6	0.413
Glycemia (mg/dl)	52	82.7	19.5	54	89.0	11.5	
IgG (mg/dl)	52	227.7	126.4	0	–	–	–
IgA (mg/dl)	52	9.1	13.4	0	–	–	–
IgM (mg/dl)	52	22.7	48.5	0	–	–	–
Total proteins (g/dl)	52	6.6	0.5	33	7.5	0.5	**<0.0001**
Alpha2 (%)	52	11.7	2.1	0	–	–	–
Iron (μg/dl)	52	64.4	29.8	36	96.3	42.0	**<0.0001**
Ferritin (ng/ml)	52	127.1	176.9	15	110.8	69.5	0.412
Fibrinogen (mg/dl)	51	355.4	91.0	0	–	–	–
C reactive protein (mg/dl)	51	0.6	0.6	11	0.4	0.1	1
ESR (mm)	52	12.8	10.7	11	11.2	8.2	0.913

*Statistically relevant values are reported in bold*.

### Western Blot Analysis

Five micrograms of serum total proteins were processed as previously described (15). All samples were tested twice in duplicate. Acrp30 antibody incubation was performed as previously described (36). Blots were developed by enhanced chemiluminescence (Amersham Bio-sciences) with Kodak BioMax Light film, digitalized with a scanner (1,200 dpi) and analyzed by densitometry with the ImageJ software (http://rsbweb.nih.gov.ij/). All experiments were performed in triplicate.

### Gel Filtration Analysis

We analyzed the distribution of Acrp30 oligomers in serum by fast protein liquid chromatography (FPLC) on a Superdex 200 10/300 GL column connected to a FPLC system (Amersham Pharmacia Biotech, Sweden). In detail, 1.875 mg of total proteins was fractionated at 0.5 ml/min using phosphate-buffered saline elution buffer. Fractions (500 µl) were collected and Acrp30 was tested using ELISA (70 µl) and western blotting (20 µl) assays. The column was calibrated using ferritin (440 kDa), aldolase (158 kDa), and ovalbumin (44 kDa) (GE Healthcare).

### Statistical Analysis

Data are expressed as means ± SD and median. The significances of biochemical parameters differences were determined using the Mann–Whitney test. The chi-square test was used to compare sex ratio. A multiple logistic regression analysis was performed to correct the significant *p*-values of the univariate analysis. We performed a receiver operating characteristic curve (ROC) analysis to determine whether Acrp30 levels are specifically modulated in CVID and the optimum cutoff point. The one-way repeated measures analysis of variance with Greenhouse–Geisser corrections was used to examine the association among biochemical parameters, Acrp30, and IgG levels after replacement therapy. Pairwise comparisons were performed using Fisher’s Least Significant Difference method. The statistical significance was established at *p* < 0.05.

## Results

### Anthropometric and Biochemical Features

**Figure 1:**
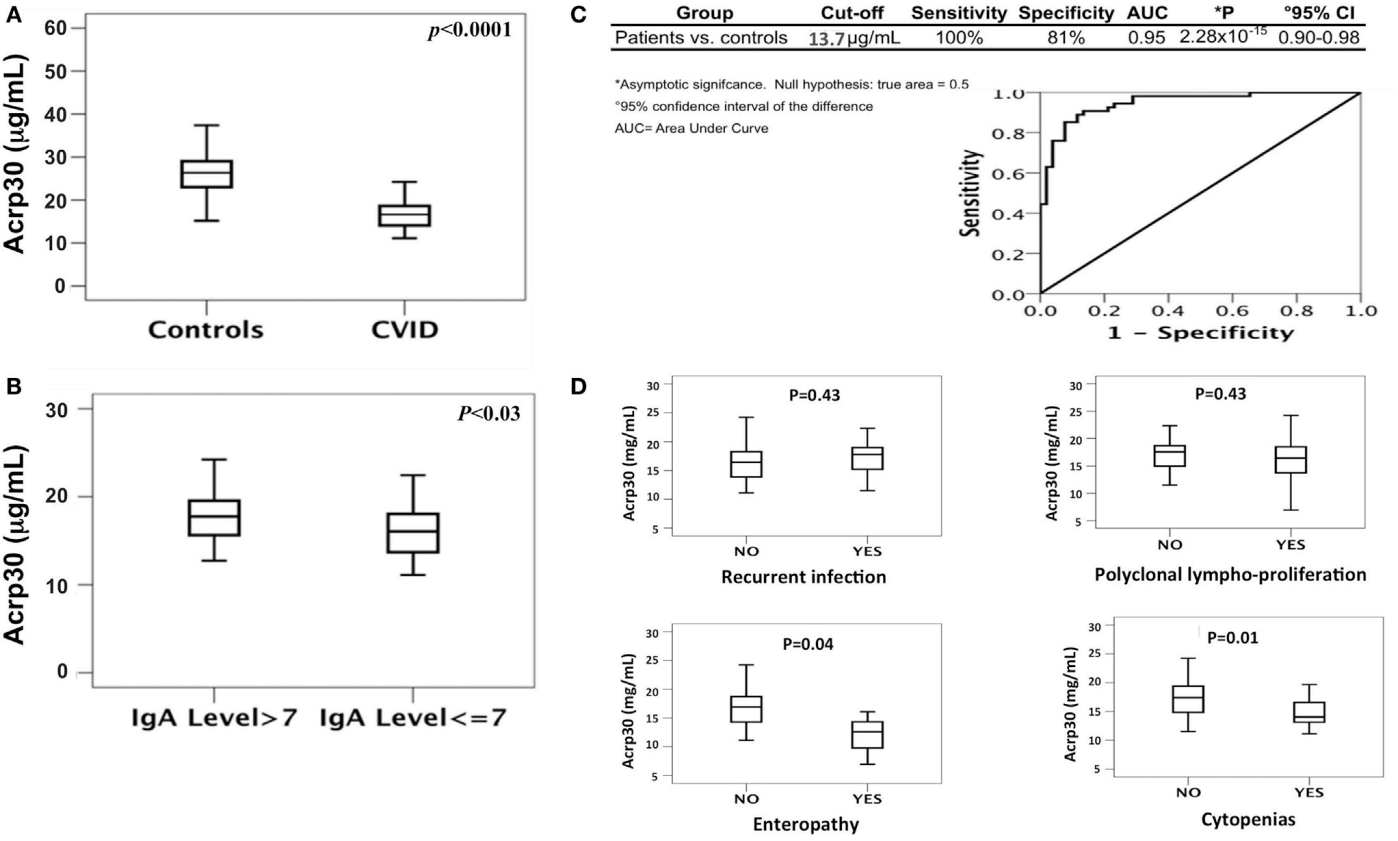
Total adiponectin (Acrp30) levels are reduced in common variable immunodeficiency (CVID) patients compared to controls. **(A)** Box plots of Acrp30 serum levels in 52 CVID patients compared to 54 controls. **(B)** Box plots of Acrp30 serum levels in 52 CVID patients stratified according to IgA levels (7 g/l > IgA > 7 g/l). **(C)** Receiver operating characteristic curves of Acrp30 serum levels: values of CVID patients, controls, and graphical representation of CVID patients compared to controls. **(D)** Box plots of Acrp30 serum levels in CVID patients stratified according to clinical variables (recurrent infections, polyclonal lymphoproliferation, enteropathy, and cytopenias). For other details, see Section “Materials and Methods.”

As shown in Table 1, the levels of total cholesterol (*p* < 0.015), total proteins (*p* < 0.0001), and iron (*p* < 0.0001) were lower in CVID patients than in controls. Both SDS-PAGE and western blotting analysis revealed the absence of Acrp30 in Ig solutions. Furthermore, cross-reactivity between Acrp30 antibodies and Ig was excluded by testing two different concentrations of Ig (6 and 8 mg/ml) in ELISA, whereas the level of Acrp30 was significantly lower in controls than in CVID patients (17 ± 0.4 versus 27 ± 0.7 µg/ml; *p* < 0.0001) (Figure 1A). After correcting for iron and cholesterol covariates, total Acrp30 levels remained significantly lower in CVID patients (*p* < 0.0001). Moreover, Acrp30 levels were higher in the subgroup of patients with an IgA level at diagnosis >7 mg/dl than in those with an IgA level at diagnosis ≤7 mg/dl (1.8 ± 0.3 versus 1.6 ± 0.3 µg/ml; *p* = 0.03) (Figure 1B).

### Acrp30 Expression in CVID Patients Compared to Controls

We performed a ROC curve analysis to verify if Acrp30 level could be an independent discriminative value to predict CVID (Figure 1C). The comparison between 54 controls and 52 CVID patients demonstrated that a cutoff value of 13.7 µg/ml Acrp30 corresponded to a sensitivity of 100% and a specificity of 81% (AUC = 0.947, *p* = 2.28 × 10^−15^). Using this cutoff point, Acrp30 levels were significantly lower in the “autoimmune cytopenias” and “enteropathy” subsets than in the controls, whereas no differences versus controls were found in the “polyclonal lymphoproliferation” and “recurrent infections” subsets (Figure 1D).

### Western Blotting and FPLC Analysis of Acrp30 Oligomerization State

**Figure 2:**
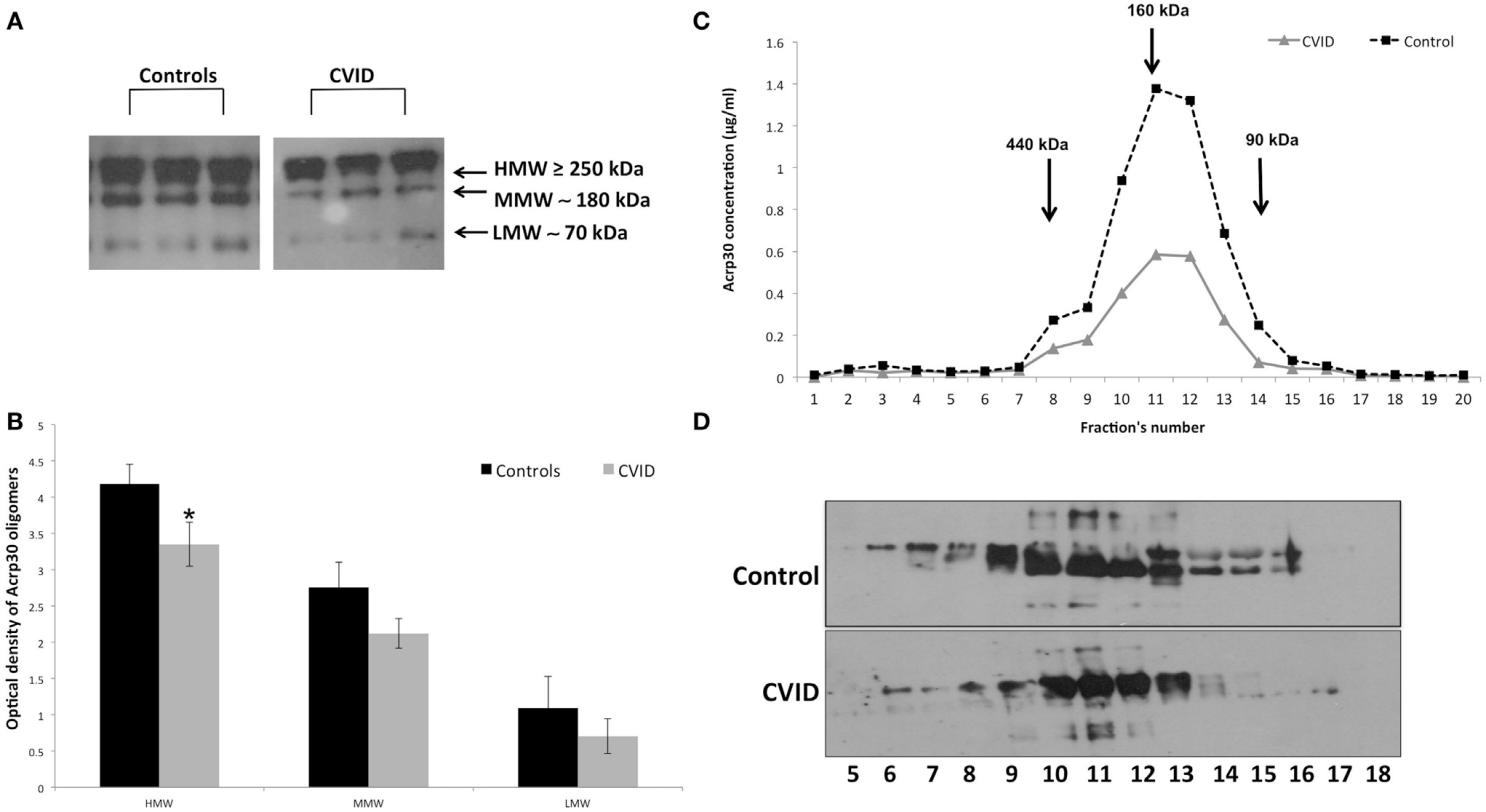
Adiponectin (Acrp30) high molecular weight (HMW) oligomers are strongly reduced in common variable immunodeficiency (CVID) patients compared to controls. **(A)** One representative western blotting image of Acrp30 oligomers [HMW, medium molecular weight (MMW), low molecular weight (LMW)] in serum of three controls, and three CVID patients. **(B)** Graphical representation of pixel quantization of all controls and CVID patients analyzed through western blotting. Acrp30 concentration in each fraction obtained from fast protein liquid chromatography analysis and subjected to **(C)** enzyme-linked immunosorbent assay assay and **(D)** western blotting. For other details see Section “Materials and Methods” (*p < 0.05).

Figure 2 shows the distribution of serum Acrp30 in control subjects and in CVID patients. After WB analysis performed on serum, three bands corresponding to HMW (≥250 kDa), MMW (≥180 kDa), and LMW (≥70 kDa) oligomers were identified in both controls and CVID patients (Figure 2A). As shown in Figure 2B, the expression of the HMW oligomers was significantly lower in CVID patients than in controls (*p* < 0.05). We also examined the distribution of Acrp30 oligomers by FPLC under native conditions. Both ELISA analysis (Figure 2C) and western blotting (Figure 2D) confirmed that Acrp30 levels are lower in CVID patients than in controls. Notably, HMW were the oligomers predominantly reduced.

### Total Acrp30 and HMW Oligomer Levels Were Significantly Higher after IVIG Replacement Therapy in Treatment-Naïve CVID Patients but Not in CIPD Patients

To investigate whether Acrp30 levels are related to Ig replacement therapy, we measured Acrp30 levels in eight treatment-naïve CVID patients before and after the first Ig replacement therapy, i.e., at 0, 1, 7, 14, and 21 days. Interestingly, Acrp30 levels were greatly increased already 24 h (208% ± 7.45) after Ig infusion in all patients and remained high up to 21 days posttreatment and declined thereafter. Table 2 shows the relationship between Acrp30 and IgG levels, pre- and post-replacement therapy in eight treatment-naïve patients. A one-way repeated measures analysis of variance with Greenhouse–Geisser corrections showed that Acrp30 and IgG levels were significantly associated with the treatment at five time points (*p* = 0.003 and *p* = 0.000003). The mean difference in Acrp30, IgA, and IgG levels between time 0 and the other time points was high but not significant, probably due the low number of patients (Table 2).

**Table 2:** Adiponectin (Acrp30) levels strongly increase in common variable immunodeficiency (CVID) naïve patients but not patients with CIDP after Ig replacement therapy.

Time (days)						
Parameters	0	1	7	14	21	*p*-Value
Acrp30 in CVID naïve patients (μg/ml)	10.96 ± 7.63	23.11 ± 6.82	21.82 ± 3.56	20.14 ± 2.65	17.65 ± 4.34	0.003
IgG in CVID naïve patients (g/l)	2.79 ± 1.98	8.79 ± 2.02	6.50 ± 1.65	6.49 ± 1.89	5.37 ± 2.32	0.000003
Acrp30 (μg/ml) in CIDP	15.28 ± 1.48	17.27 ± 0.32	17.28 ± 0.29	nd	nd	>0.05
IgG (g/l) in CIDP	11.75 ± 1.94	23.66 ± 2.36	17.705 ± 4.21	nd	nd	0.004

*Data given are mean ± SD of eight CVID naïve patients and five patients with chronic inflammatory demyelinating polyneuropathy (CIDP)*.

*p-Value is obtained by one-way repeated measures analysis of variance with Greenhouse–Geisser corrections*.

To verify if the Acrp30 increase is a specific characteristic of CVID patients subjected to Ig replacement therapy, we evaluated its levels before, and 24 h and 7 days after Ig immunomodulating therapy in CIDP patients, and found that the total Acrp30 level did not change (see Table 2). The specificity of the Acrp30 response to Ig administration is guaranteed by the high level of purity (>99%) of the Ig solution injected in patients. We excluded Acrp30 contamination in Ig preparation by SDS-PAGE and Coomassie coloration or western blot analysis (data not shown).

Furthermore, cross-reactivity between Acrp30 antibodies and Ig in ELISA was excluded by testing as controls two different concentrations of Ig (6 and 8 mg/ml). Each serum sample was tested four times in triplicate.

**Figure 3:**
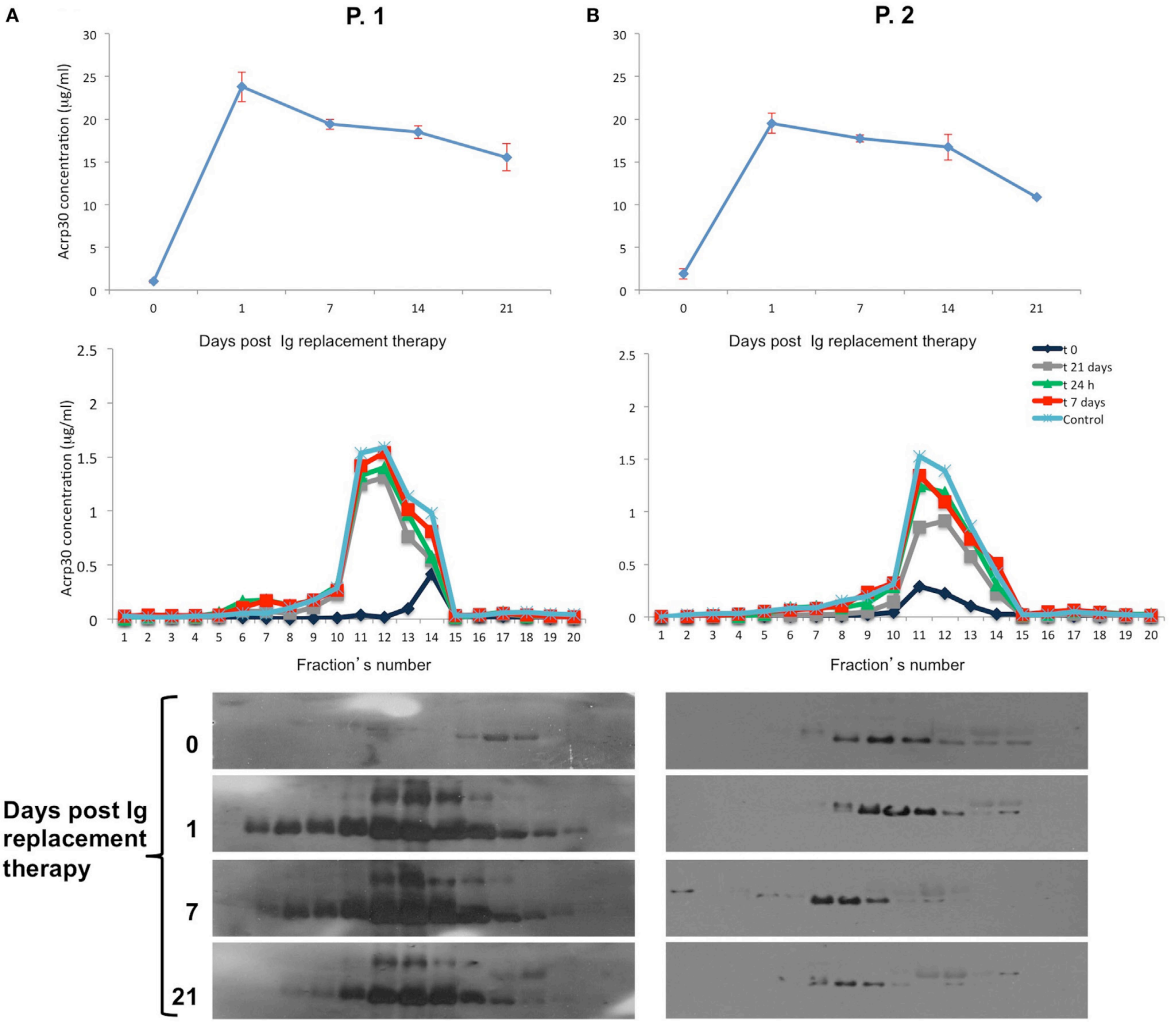
Adiponectin (Acrp30) levels strongly increase after Ig replacement therapy in common variable immunodeficiency (CVID) treatment-naïve patients. Acrp30 and high molecular weight oligomers in two representative CVID naïve patients P-1 and P-2 subjected to Ig replacement therapy. **(A)** Total Acrp30 serum levels obtained through enzyme-linked immunosorbent assay (ELISA) assay; Acrp30 oligomers distribution in each fraction obtained from fast protein liquid chromatography analysis and subjected to ELISA assays **(B)** and western blotting **(C)**. The patients have been analyzed before starting treatment (naïve = 0) and during the time course post therapy (1, 7, 14, and 21 days). For other details, see Section “Materials and Methods.”

Table S1 in Supplementary Material shows the biochemical and anthropometrical parameters of 4 males and four females treatment-naïve patients. Ig administration strongly increased Acrp30 levels as early as 24 h after infusion in both patients, and remained high up to 14 days posttreatment; they were slightly decreased 21 days after treatment (Figure 3, upper panel). As expected, IgG levels were very high 24 h after treatment and remained high up to 21 days.

## Discussion

The molecular mechanism underlying the clinical manifestations of CVID has not yet been completely elucidated. However, chronic activation of the immune system in CVID patients is accompanied by dysregulation of various inflammatory cytokines (37–39), and an intricate adipokine-related network links adipose tissue to the immune system (34). In the attempt to shed light on Acrp30 in the adipokine-related network in CVID, we characterized the expression of Acrp30 and HMW oligomers, the most active forms, in patients undergoing maintenance Ig replacement therapy as well as in treatment-naïve CVID patients (11, 15, 32). We found that the levels of Acrp30 and its HMW oligomers were lower in CVID patients than in age- and sex-matched controls, and that they correlate with IgA levels in these patients. Acrp30 levels were significantly lower in the subset of CVID patients with IgA levels at diagnosis ≤7 mg/dl than in those with IgA levels at diagnosis >7 mg/dl, which confirms the strict correlation between adipose tissue and the immune system. Quinti et al. reported that IgA levels at diagnosis <7 mg/dl define a subgroup of patients at a higher risk for pneumonia and, consequently, a poor prognosis (40). Similarly, we show that a late diagnosis is associated with a worst prognosis in CVID patients with IgA serum levels below 7 mg/dl (41). In this scenario, the very low serum expression of Acrp30 in patients with the most CVID severe phenotypes suggests that this adipokine could be associated to some clinical manifestations of CVID and could thus be considered a potential marker of the severity and prognosis of this disease.

In the light of this evidence, we investigated the effects of Ig replacement on Acrp30 expression in treatment-naïve CVID patients before and after the first Ig administration. Interestingly, in these patients, Ig replacement therapy rapidly induced an increase of Acrp30, which was associated with IgG levels. On the contrary, Acrp30 levels did not change after Ig replacement therapy in patients affected with a non-immunodeficiency syndrome, namely, CIDP, which indicates that Acrp30 plays a role in CVID. One-way repeated analysis showed a direct relationship between Acrp30 and IgG levels (*p* = 0.09 and *p* = 0.087) at two time points in the treatment-naïve patients thereby confirming the specificity of the relationship between the increase in Acrp30 and Ig administration.

Our data suggest that adipose tissue functions as an endocrine organ and participates in the immune response. In addition, our analysis of CIPD patients confirmed the specificity of Acrp30 expression in response to Ig therapy in CVID patients. In fact, Acrp30 levels did not increase in these patients after the administration of either 0.4 g/kg of Ig (24 h posttreatment) or 2 g/kg of Ig (7 days posttreatment). These findings indicate that Ig administration *per se* is not able to induce the increase of Acrp30, but it seems that the specific cellular and/or molecular background of CVID is required to modulate Acrp30 levels. However, a limitation of our study is the relatively low number of CIDP patients. Since our treatment-naïve CVID patients were characterized by low Acrp30 concentrations (Table 2), we hypothesize that Ig administration modulates the activation state and the cytokine profile involved in the chronic immune activation signature of CVID. Therefore, the increase of Acrp30 levels observed in treatment-naïve CVID patients could be related to the change in the cytokine milieu induced by Ig administration. In this context, it is noteworthy that Ig, greatly reduced the expression of the pro-inflammatory IL-1b and IL-6 cytokines in adipocytes (42, 43).

We also found an association between Acrp30 and autoimmune cytopenias/enteropathy phenotypes in the CVID cohort. In detail, Acrp30 levels were significantly lower in patients with autoimmune cytopenias/enteropathies than in patients with other phenotypes. This finding is relevant considering that about 10–20% of CVID patients suffer from autoimmune cytopenias (2, 44–47) and 10% from enteropathy (2). Moreover, CVID patients without infectious complications have a poorer prognosis than the “infections only” subset, and have an 11-fold higher risk of death (40). On the other hand, CVID patients with enteropathies very often have a local or systemic inflammatory state characterized by elevated levels of pro-inflammatory cytokines that could contribute to the decrease of Acrp30 levels (48). Thus, the dysregulation of Acrp30 expression we observed could be related to the biological mechanisms in which this adipokine is involved, i.e. inflammatory processes and immune cell regulation (49). On the other hand, dysregulated Acrp30 levels have been found in such immune disorders as rheumatoid arthritis (50), systemic lupus erythematosus (51), and inflammatory bowel disease (52). Whether Acrp30 is an anti- or pro-inflammatory factor remains to be established, even though the prevailing notion is that it is a pro-inflammatory molecule (31, 53, 54). Consistent with this concept, adiponectin knockout mice display increased M1 markers and decreased M2 markers (55–57). Notably, Acrp30, by reducing T cell transmigration across the endothelium, functions as an immune suppressor molecule (22, 24, 58, 59). It activates plasma B cells and induces secretion of the B cell-derived peptide PEPITEM, which inhibits memory T cell migration (60).

In conclusion, the correlations between Acrp30, biological markers and severe CVID phenotypes indicate that Acrp30 plays a central role in the immune activation typical of this disease. Further studies are needed to clarify the molecular mechanisms underlying Acrp30 regulation and to decipher the biological role of this adipokine in the immune system.

## Ethics Statement

The research protocol was approved by the Ethics Committee of the School of Medicine, University of Naples “Federico II” and was in accordance with the principles of the Helsinki II Declaration.

## Author Contributions

AD, GS, and AG substantially conceived the research and directed and discussed the comprehensive assembly of data acquisition, analysis, and interpretation; AP, IM, and ACM recruited, treated, and followed up CVID patients; FH recruited and treated CIDP patients; MC performed the statistical analysis; EN, RP, and MM did most of experimental work; EN and OS performed and coordinated the laboratory experimental and data acquisition ensuring that questions related to the accuracy or integrity of any part of the work are appropriately investigated and resolved; AD, GS, and AG, with AP and EN, wrote the manuscript and give the final approval for publication.

## Conflict of Interest Statement

The authors declare that the research was conducted in the absence of any commercial or financial relationships that could be construed as a potential conflict of interest.

## Abbreviations

Acrp30, adiponectin; APN-KO, adiponectin knockout; BMI, body mass index; COPD, chronic obstructive pulmonary disease; CRP, C reactive protein; CVID, common variable immunodeficiency; ELISA, enzyme-linked immunosorbent assay; FPLC, fast protein liquid chromatography; HMW, high molecular weight; Ig, immunoglobulin; IgA, immunoglobulin A; IgG, immunoglobulin G; IL-6, interleukin 6; IVIG, intravenous immunoglobulin; LMW, low molecular weight; MMW, medium molecular weight; NF-κB, nuclear factor kappa-light-chain-enhancer of activated B cells; OSAS, obstruction sleep apnea syndrome; PBMCs, peripheral blood mononuclear cells; ROC, receiver operating characteristic; SDS-PAGE, sodium dodecyl sulfate-polyacrylamide gel electrophoresis; TNF-α, tumor necrosis factor α.
