# Supplementary material for: Total and High Molecular Weight Adiponectin Expression Is Decreased in Patients with Common Variable Immunodeficiency: Correlation with Ig Replacement Therapy
Source: Front Immunol. 2017 Jul 31;8:895. doi: 10.3389/fimmu.2017.00895 (PMC5534466; doi:10.3389/fimmu.2017.00895)
Supplement: Supplementary file 1 [file Table_1.docx]

**Supplementary Table 1. Clinical and biochemical features of CVID naïve patients, pre- and post- Ig replacement therapy.**

|  | **CVID Patients (4 males)** | | | | | **CVID Patients (4 females)** | | | | |
| --- | --- | --- | --- | --- | --- | --- | --- | --- | --- | --- |
| **Parameters** | **naive** | **t**  **24h** | **t**  **7days** | **t**  **14days** | **t**  **21days** | **naive** | **t**  **24h** | **t**  **7days** | **t**  **14days** | **t**  **21days** |
| Sex | M | M | M | M | M | F | F | F | F | F |
| Age (years) | 38±16.39 | 38±16.39 | 38±16.39 | 38±16.39 | 38±16.39 | 40.7±24.25 | 40.7±24.25 | 40.7±24.25 | 40.7±24.25 | 40.7±24.25 |
| Weight (kg) | 62.43±2.47 | 62.43±2.47 | 62.56±2.47 | 62.5±2.47 | 62.5±2.47 | 72±18.52 | 70±15.52 | 70±15.52 | 70±15.52 | 70±15.52 |
| BMI (kg/m^2^) | 23.07±2.47 | 23.07±2.47 | 23.15±2.46 | 23.12±2.51 | 23.10±2.43 | 24.92±4.22 | 24.25±3.12 | 24.25±3.12 | 24.5±3.12 | 23.1±2.59 |
| Total Cholesterol (mg/dl) | 190.5±17.31 | 175.5±29.08 | 162±20.88 | 182.66±42.06 | 172±30.80 | 167.5±47.05 | 162.5±39.26 | 160.66±35.50 | 153±34.00 | 149±34.21 |
| Tryglicerides (mg/dl) | 82.75±19.08 | 60.5±6.45 | 84.33±8.96 | 120.66±68.06 | 79.66±25.00 | 124.75±27.21 | 96±24.67 | 97±28.68 | 82.66±12.09 | 87.33±12.58 |
| Glycemia (mg/dl) | 79.75±6.18 | 79.75±6.55 | 73±5.56 | 71.66±10.26 | 67.33±5.51 | 85.75±14.08 | 88.75±8.46 | 86±4.58 | 84.66±3.21 | 82.33±4.04 |
| IgG (g/l) | 2.24±2.43 | 8.71±0.84 | 5.86±0.92 | 5.94±1.31 | 4.52±2.13 | 3.05±1.11 | 8.86±2.95 | 7.13±2.10 | 7.02±2.41 | 6.22±2.46 |
| IgA (g/l) | 1.5±0.57 | 1.25±0.50 | 1.25±0.50 | 1.25±0.50 | 1.75±0.50 | 1.25±0.5 | 1±0.0 | 1±0.0 | 1±0.0 | 1±0.0 |
| IgM (g/l) | 0.407±0.48 | 0.386±0.46 | 0.318±0.31 | 0.378±0.44 | 0.346±0.47 | 0.26±0.09 | 0.29±0.09 | 0.30±0.13 | 0.31±0.14 | 0.32±0.13 |
| Total Proteins (g/dl) | 6.32±1.40 | 6.47±0.75 | 6.06±0.35 | 6.1±0.43 | 5.76±0.11 | 5.75±0.86 | 6.3±0.82 | 6.5±0.45 | 6.56±0.25 | 6.36±0.11 |
| Alpha2 (%) | 12.3±2.68 | 11.27±1.80 | 11.4±0.43 | 11.03±1.96 | 10.83±1.43 | 10.55±3.02 | 10.67±3.28 | 9.53±0.60 | 9.8±0.62 | 9.63±0.65 |
| Iron (µg/dl) | 61.5±34.34 | 54.25±34.75 | 61.66±22.00 | 49.00±6.93 | 54.66±17.61 | 43±26.24 | 28.25±13.69 | 34.33±16.16 | 34.66±8.08 | 34.66±8.08 |
| Ferritin (ng/ml) | 183±107.75 | 122.25±54.78 | 112±73.81 | 98.66±67.42 | 102.66±63.28 | 71.25±50.10 | 75.5±49.74 | 69.0±51.97 | 68.66±51.67 | 69.33±51.39 |
| Fibrinogen (mg/dl) | 321.67±57.17 | 311.85±37.37 | 314.34±9.38 | 312.48±82.64 | 276.55±61.53 | 296.75±134.94 | 299.44±94.96 | 255.06±65.42 | 268.38±86.60 | 273.66±71.43 |
| CRP (mg/dL) | 1.34±1.53 | 0.485±0.25 | 0.473±0.24 | 0.54±0.29 | 0.48±0.13 | 1.43±2.2 | 0.89±1.04 | 0.37±0.07 | 1.17±1.46 | 1.17±1.46 |
| ESR (mm) | 11.50±7.85 | 9.75±4.78 | 12.00±4.58 | 7.00±2.64 | 12.00±8.71 | 9±12.78 | 11.5±17.69 | 4.33±3.21 | 4.66±5.03 | 5.66±3.78 |
